# Prevalence, expenditures, and associated factors of purchasing non-prescribed Chinese herbal medicine in Taiwan

**DOI:** 10.1371/journal.pone.0240311

**Published:** 2020-10-26

**Authors:** Feng-Ping Hu, Chien-Chan Liao, Ta-Liang Chen, Chun-Chieh Yeh, Luwen Shi, Chun-Chuan Shih

**Affiliations:** 1 School of Pharmaceutical Sciences, Peking University, Beijing, China; 2 Department of Anesthesiology, Taipei Medical University Hospital, Taipei, Taiwan; 3 Anesthesiology and Health Policy Research Center, Taipei Medical University Hospital, Taipei, Taiwan; 4 Department of Anesthesiology, School of Medicine, College of Medicine, Taipei Medical University, Taipei, Taiwan; 5 Research Center of Big Data and Meta-Analysis, Wan Fang Hospital, Taipei Medical University, Taipei, Taiwan; 6 School of Chinese Medicine, College of Chinese Medicine, China Medical University, Taichung, Taiwan; 7 Department of Anesthesiology, Wan Fang Hospital, Taipei Medical University, Taipei, Taiwan; 8 Department of Surgery, China Medical University Hospital, Taichung, Taiwan; 9 Department of Surgery, University of Illinois, Chicago, Illinois, United States of America; 10 School of Chinese Medicine for Post-Baccalaureate, College of Medicine, I-Shou University, Kaohsiung, Taiwan; 11 Ph.D. Program in Clinical Drug Development of Herbal Medicine, College of Pharmacy, Taipei Medical University, Taipei, Taiwan; Endeavour College of Natural Health, AUSTRALIA

## Abstract

**Background and aims:**

Purchasing Chinese herbal medicine (CHM) without a physician’s prescription may have adverse effects on health. However, the recent status of purchasing non-prescribed CHM and the associated factors are not completely understood. We aimed to report the prevalence of purchasing CHM and associated factors.

**Methods:**

Using data from the 2017 Taiwan Survey of Family Income and Expenditure, we identified 16,528 individuals (householders) aged 18 years and older. Sociodemographic factors, expenditures on medical services and health behaviours were compared between people with and without a history of purchasing non-prescribed CHM by calculating adjusted odds ratios (ORs) and 95% confidence intervals (CIs) in a multiple logistic regression analysis.

**Results:**

The one-year prevalence of purchasing non-prescribed CHM was 74.8% in Taiwan. In addition to sociodemographics, marital status (OR 2.14, 95% CI 1.88–2.44), the use of traditional Chinese medicine (OR 3.62, 95% CI 3.30–3.97), the purchasing of non-prescribed biochemical medications (OR 3.09, 95% CI 2.75–3.48), the purchasing of health foods (OR 2.59, 95% CI 2.33–2.86), the use of folk therapy (OR 2.27, 95% CI 1.95–2.64), and a high level of expenditure on alcohol (OR 3.79, 95% CI 3.29–4.36) were strongly correlated with purchasing non-prescribed CHM.

**Conclusion:**

The one-year prevalence of purchasing non-prescribed CHM is very high in Taiwan and is correlated with sociodemographics, health behaviours, and the utilization of medical care. The interaction of non-prescribed CHM with physician-prescribed herbal medicine and biomedical medications requires more attention.

## Introduction

With the growing knowledge of and positive attitudes towards the self-management of health and medical care around the world, complementary alternative medicine (CAM) is increasing been widely used by people in Western and Asian countries [1–3]. It has been reported that the one-year prevalence of CAM use among the general population in Europe, the United States, and Japan was 25.9% in 2014, 33.2% in 2012, and 62.1% in 2009, respectively [1–3]. According to an estimation made based on a national survey, the total out-of-pocket expenditures for various types of complementary health approaches in the United States were as high as $30.2 billion [4].

Chinese herbal medicine (CHM) and acupuncture are the two most important treatment types in traditional Chinese medicine (TCM), which is a type of CAM. The high prevalence of the lifetime use of acupuncture (7.7%) and CHM (4.8%) in Australian women was investigated in the previous study [5]. In the United States, the prevalence of the lifetime use of acupuncture was 4.1% in 2002 and increased to 6.8% in 2007 among adults aged 18 years and over [6, 7]. An internet-based survey showed that the one-year prevalence of TCM use was as high as 39.7% in 2018 in Taiwan [8]. TCM has beneficial effects among patients with various diseases, such as stroke, cancer, pain and allergic rhinitis [9–12]. Although the outpatient payments for TCM have been covered by Taiwan’s National Health Insurance Program since 1995, it is still common for people to purchase CHM without a physician’s prescription in traditional pharmacies or illegal markets [13, 14].

Herb-drug interactions, unqualified herbal medicine, and purchasing non-physician-prescribed CHM may lead to adverse effects, such as serotonin syndrome, rhabdomyolysis, acute delirium [15–18]. Although the prevalence and characteristics of and the factors associated with purchasing non-prescribed CHM among adults in Taiwan have been reported, there were several limitations of these studies, such as the use of outdated data (surveys performed in 2005 or 2006) [13, 14], a small sample size (641 participants) [14], a focus on short-term use (one month) [14], and the lack of CHM expenditure data [13, 14]. Therefore, there is a need for recent information regarding the people who purchase non-prescribed CHM in Taiwan. Using data from the Taiwan Survey of Family Income and Expenditure (TSFIE) in 2017, we conducted a cross-sectional study to investigate the recent prevalence and characteristics of and factors associated with the purchase of non-prescribed CHM among adults in Taiwan.

## Methods

### Source of data and study design

We used the database from the 2017 TSFIE that was conducted by the government (the Directorate-General of the Budget, Accounting and Statistics, Executive Yuan, Taiwan). The sources of the data, sampling methods, and measurement details were described in a previous study [19]. In brief, the researchers used a stratified random sampling method to choose the sampled households from the whole population in Taiwan, and these households were interviewed annually to determine their major sources of income and expenditures. The householder was the representative of the family, and he or she was asked to answer questions regarding daily income and expenditure activity within the previous year. The population of Taiwan is approximately 23 million and is distributed throughout 7 cities and 18 counties. The universal sampling rate was approximately 0.20%, with 16,528 sampled households. The survey content is categorized into four domains: household members, household facilities and housing conditions, income and expenditures, and consumption expenditure. The 2017 TSFIE covers the one-year period from January 1 to December 31, and the interview was administered from January 1 to February 28 in 2018. At the beginning of each interview, the interviewer explained the survey purpose and asked if the interviewee was an income earner aged 20 years or over who lived in the household. Because the 2017 TSFIE was administered by face-to-face interviews with account-keeping collection methods, it was more accurate than studies conducted solely based on interviews.

Using the data from the 2017 TSFIE, we conducted this cross-sectional study to investigate the prevalence of purchasing CHM without a physician’s prescription and the associated factors. Information from the 2017 TSFIE database used in this study included a series of personal and family characteristics, including sociodemographics (age, sex, marital status, education level, employment status, and geographic locations), health care utilization, individual and household out-of-pocket expenditures, etc.

### Ethical approval

At the initial interview during the 2017 TSFIE, the participants were asked for permission for access to the interviewed database for research purposes, and all study participants signed the informed consent form. To protect personal privacy, the electronic database was encoded, and the identifiers of participants were scrambled prior to further academic access for research. The current study was also evaluated and approved by the Directorate-General of Budget, Accounting and Statistics, Executive Yuan, Taiwan and the Joint Institutional Review Board of E-DA Hospital (EDA-JIRB-2017002; EDA-JIRB-2018008) and Taipei Medical University (TMU-JIRB-202003097).

### Definition and measures

In Taiwan, TCM is legal, and it is covered by the National Health Insurance Program for ambulatory care but not inpatient care. TCM practitioners are registered TCM doctors who have been certified by the Ministry of Health and Welfare, and they can only provide TCM in a hospital or clinic. Western medicine doctors cannot practise TCM in Taiwan. According to medical law in Taiwan, TCM doctors can advertise the medical benefits of TCM. TCM includes herbal medicine, acupuncture, moxibustion, bone reduction, traditional trauma treatment, traditional dislocation treatment, traditional fracture treatment, Tuina, Baguan, and other therapies. However, not all TCM modalities are covered by the National Health Insurance.

The core question in the 2017 TSFIE was “Have you ever purchased Chinese herbal medicine for yourself or your family members without a physician’s prescription in the past year?” In this study, we defined purchasing non-prescribed CHM as purchasing CHM without a physician’s prescription. The purchased non-prescribed CHM included Chinese medicine materials (such as Dang Guei, Ren Shen, Sih-Wu-Tang, Si Shen Tang, Shi Quan Da Bu Tang, Gou Ci Zih), tinctures, traditional formulas, and concentrated preparations. People can purchase non-prescribed CHM from biochemical pharmacies (such as over-the-counter CHM, Good Manufacturing Practice products), CHM pharmacy (such as Chinese medicine materials and concentrated preparations), and illegal markets.

### Statistical analyses

We used chi-square tests to compare the differences in sociodemographic factors (such as age, sex, occupation, education, marital status, level of income, urbanization), lifestyle factors (including smoking and alcohol consumption), and medical care behaviours (receiving Western medical care, TCM and dental care) between people who had and had not purchased non-prescribed CHM.

The crude odds ratios (ORs) and 95% confidence intervals (CIs) of factors associated with purchasing non-prescribed CHM were calculated by univariate logistic regression. These factors included age, sex, occupation, education, marital status, level of income, urbanization, smoking expenditure, alcohol expenditure and the use of Western medicine, TCM and dental care. The significant factors (p<0.05) were then entered into multivariate logistic regression analysis to calculate the adjusted ORs and 95% CIs of the factors associated with purchasing non-prescribed CHM.

For each covariate, we assigned a predictive score as a risk index according to the significant adjusted OR, and thepredictive score was proportional to the OR. The purchasing predictive score was defined as follows: when 1.0 ≤ OR<1.5, the purchasing predictive score was 1; when 1.5 ≤ OR <2.0, the purchasing predictive score was 2; when 2.0 ≤ OR <2.5, the purchasing predictive score was 3; and when 2.5 ≤ OR <3.0, the purchasing predictive score was 4. All analyses were performed using Statistical Analysis Software (SAS), version 9.2 (SAS Institute Inc., Cary, North Carolina, USA). A two-sided p-value less than 0.05 was considered statistically significant.

## Results

The one-year prevalence of purchasing non-prescribed CHM was 74.8% among 16,528 adults aged older than 18 years (Table 1). A higher percentage of middle-aged people (50–59 years) purchased non-prescribed CHM than people aged 18–29 years (p<0.0001), and more males purchased non-prescribed CHM than females (p<0.0001). Higher percentages of people who had an occupation in the agriculture/forestry/fishery industry, were currently married, and had high level of income purchased non-prescribed CHM than their counterparts.

**Table 1:** Baseline characteristics of people who did and did not purchase of non-prescribed Chinese herbal medicine.

	CHM purchase				*p*-Value
	No (N = 4154)		Yes (N = 12,374)		
Age, years	n	(%)	n	(%)	<0.0001
18–29	200	(29.9)	468	(70.1)	
30–39	705	(25.5)	2056	(74.5)	
40–49	985	(25.7)	2853	(74.3)	
50–59	970	(23.5)	3161	(76.5)	
60–69	710	(23.6)	2303	(76.4)	
70–97	582	(27.5)	1531	(72.5)	
Sex					<0.0001
Female	1323	(27.2)	3542	(72.8)	
Male	2831	(24.3)	8832	(75.7)	
Urbanization					<0.0001
Low	702	(30.6)	1595	(69.4)	
Moderate	1388	(22.3)	4833	(77.7)	
High	2064	(25.8)	5946	(74.2)	
Education, years					<0.0001
0	125	(34.1)	242	(65.9)	
1–9	2388	(24.5)	7343	(75.5)	
10–12	1336	(24.9)	4030	(75.1)	
≥13	305	(28.7)	759	(71.3)	
Occupation		.			<0.0001
None	1004	(30.7)	2271	(69.3)	
Agriculture/animal	164	(19.5)	676	(80.5)	
Senior technician	798	(23.7)	2573	(76.3)	
Blue collar	585	(24.0)	1853	(76.0)	
White collar	564	(24.5)	1739	(75.5)	
General service	591	(25.4)	1739	(74.6)	
Others	448	(22.7)	1523	(77.3)	
Level of income					<0.0001
Low	2132	(32.2)	4480	(67.8)	
Moderate	776	(23.5)	2529	(76.5)	
High	1246	(18.8)	5365	(81.2)	
Marital status					<0.0001
Unmarried	604	(43.0)	802	(57.0)	
Married	2288	(20.6)	8821	(79.4)	
Other	1262	(31.4)	2751	(68.6)	

CHM, Chinese herbal medicine.

As shown in Table 2, a higher prevalence of purchasing non-prescribed CHM was found among people who purchased non-prescribed biomedical drugs (p<0.0001), purchased health food (p<0.0001), spent a relatively large amount of money on alcohol, and used folk therapy (p<0.0001), dental care (p<0.0001), Western medicine (p<0.0001), and TCM (p<0.0001). Compared with the reference group in the multiple logistic regression (Table 3), the adjusted ORs for purchasing non-prescribed CHM in the groups who were 70–97 years of age, were female, had 0 years of education, and had agriculture/animal occupations were 1.51 (95% CI 1.17–1.94), 1.25 (95% CI 1.14–1.37), 1.52 (95% CI 1.10–2.10), and 1.86 (95% CI 1.51–2.30), respectively. A high level of income (OR 1.58, 95% CI 1.41–1.77), living in a highly urbanized area (OR 1.58, 95% CI 1.40–1.77), being married (OR 2.14, 95% CI 1.88–2.44), using Western medicine (OR 1.50, 95% CI 1.12–2.01), using TCM (OR 3.62, 95% CI 3.30–3.97), and not being hospitalized (OR 1.25, 95% CI 1.11–1.41) were factors associated with purchasing non-prescribed CHM. In the multiple logistic regression adjusted for covariates, the significant factors associated with purchasing non-prescribed CHM were purchasing non-prescribed biochemical medications (OR 3.09, 95% CI 2.75–3.48), purchasing health food (OR 2.59, 95% CI 2.33–2.86), using folk therapy (OR 2.27, 95% CI 1.95–2.64), and spending a relatively large amount on on alcohol (OR 3.79, 95% CI 3.29–4.36).

**Table 2:** Health management behaviours in people who did and did notpurchase of non-prescribed Chinese herbal medicine.

	CHM purchase				*p*-Value
	No (N = 4154)		Yes (N = 12,374)		
Hospitalization	n	(%)	n	(%)	0.1175
No	3666	(24.9)	11030	(75.1)	
Yes	488	(26.6)	1344	(73.4)	
Purchase of biomedical drugs					<0.0001
No	888	(56.2)	691	(43.8)	
Yes	3266	(21.8)	11683	(78.2)	
Use of folk therapy					<0.0001
No	3937	(27.1)	10571	(72.9)	
Yes	217	(10.7)	1803	(89.3)	
Purchase of health food					<0.0001
No	1252	(50.0)	1251	(50.0)	
Yes	2902	(20.7)	11123	(79.3)	
Expenditure on smoking, US dollars					0.2743
0	3061	(25.5)	8963	(74.5)	
1–300	197	(25.7)	571	(74.3)	
301–600	303	(22.8)	1027	(77.2)	
601–900	202	(24.4)	627	(75.6)	
≥900	391	(24.8)	1186	(75.2)	
Expenditure on alcohol, US dollars					<0.0001
0	1114	(53.5)	967	(46.5)	
1–30	1601	(24.7)	4871	(75.3)	
31–60	452	(20.1)	1793	(79.9)	
61–150	502	(15.6)	2716	(84.4)	
≥150	485	(19.3)	2027	(80.7)	
Use of dental care					<0.0001
No	1209	(35.8)	2166	(64.2)	
Yes	2945	(22.4)	10208	(77.6)	
Use of Western medicine					<0.0001
No	142	(55.9)	112	(44.1)	
Yes	4012	(24.7)	12262	(75.3)	
Use of traditional Chinese medicine					<0.0001
No	3403	(24.5)	6026	(75.5)	
Yes	751	(10.6)	6348	(89.4)	

CHM, Chinese herbal medicine.

**Table 3:** Adjusted odd ratios (95% confidence intervals) and predictive scores of factors associated with purchasing non-prescribed Chinese herbal medicine.

		CHM purchase				
		n	Rate, %	OR	(95% CI)	Scores
Age, years	18–29	668	70.1	1.00	(Reference)	0
	30–39	2761	74.5	1.01	(0.83–1.25)	0
	40–49	3838	74.3	1.01	(0.83–1.23)	0
	50–59	4131	76.5	1.07	(0.88–1.32)	0
	60–69	3013	76.4	1.28	(1.02–1.59)	1
	70–97	2113	72.5	1.51	(1.17–1.94)	2
Sex	Female	4865	72.8	1.25	(1.14–1.37)	0
	Male	11663	75.7	1.00	(Reference)	0
Education, years	0	367	65.9	1.52	(1.10–2.10)	2
	1–9	9731	75.5	1.54	(1.28–1.85)	2
	10–12	5366	75.1	1.26	(1.06–1.49)	1
	≥13	1064	71.3	1.00	(Reference)	0
Occupation	None	3275	69.3	1.00	(Reference)	0
	White collar	2303	75.5	0.94	(0.79–1.14)	0
	Senior technician	3371	76.3	1.06	(0.90–1.25)	0
	Services	2330	74.6	1.07	(0.91–1.25)	0
	Agriculture/animal	840	80.5	1.86	(1.51–2.30)	2
	Blue collar	2438	76.0	1.29	(1.09–1.52)	1
	Others	1971	77.3	1.15	(0.96–1.38)	0
Level of income	Low	6612	67.8	1.00	(Reference)	0
	Moderate	3305	76.5	1.27	(1.13–1.42)	1
	High	6611	81.2	1.58	(1.41–1.77)	2
Urbanization	Low	2297	69.4	1.00	(Reference)	0
	Moderate	6221	77.7	1.27	(1.13–1.42)	1
	High	8010	74.2	1.58	(1.40–1.77)	2
Marital status	Unmarried	1406	57.0	1.00	(Reference)	0
	Married	11109	79.4	2.14	(1.88–2.44)	3
	Other	4013	68.6	1.52	(1.32–1.75)	2
Use of dental care	No	3375	64.2	1.00	(Reference)	0
	Yes	13153	77.6	1.05	(0.95–1.16)	0
Use of WM	No	254	44.1	1.00	(Reference)	0
	Yes	16274	75.3	1.50	(1.12–2.01)	2
Use of TCM	No	9429	75.5	1.00	(Reference)	0
	Yes	7099	89.4	3.62	(3.30–3.97)	5
Hospitalization	No	14696	75.1	1.25	(1.11–1.41)	1
	Yes	1832	73.4	1.00	(Reference)	0
Purchase of medications	No	1579	43.8	1.00	(Reference)	0
	Yes	14949	78.2	3.09	(2.75–3.48)	5
Purchase of health food	No	2503	50.0	1.00	(Reference)	0
	Yes	14025	79.3	2.59	(2.33–2.86)	4
Use of folk therapy	No	14508	72.9	1.00	(Reference)	0
	Yes	2020	89.3	2.27	(1.95–2.64)	3
Smoking expenditure	No	12024	74.5	1.24	(1.08–1.41)	1
	1–300	768	74.3	0.98	(0.79–1.23)	0
	301–600	1330	77.2	1.24	(1.03–1.50)	1
	601–900	829	75.6	1.11	(0.75–1.08)	0
	≥900	1577	75.2	1.00	(Reference)	0
Alcohol expenditure	No	2081	46.5	1.00	(Reference)	0
	1–30	6472	75.3	2.59	(2.32–2.90)	4
	31–60	2245	79.9	2.99	(2.59–3.46)	4
	61–150	3218	84.4	3.79	(3.29–4.36)	5
	≥150	2512	80.7	3.04	(2.62–3.53)	5

CHM, Chinese herbal medicine; CI, confidence interval; OR, odds ratio; WM, Western medicine.

Compared with people with a predictive score from 4 to 10 (Table 4), the adjusted ORs of purchasing non-prescribed CHM for those with scores from 11 to 15, from 16 to 20, from 21 to 25, from 26 to 30, and ≥31 were 1.97 (95% CI 1.34–2.89), 4.28 (95% CI 2.98–6.15), 10.7 (95% CI 7.50–15.4), 41.4 (95% CI 28.7–59.7), and 121 (95% CI 77.4–189), respectively.

**Table 4:** Predictive scores associated with purchasing non-prescribed Chinese herbal medicine.

		CHM purchase			
	n	Users	Rate, %	OR	(95% CI)
Predictive score					
4–10	194	38	19.6	1.00	(Reference)
11–15	821	266	32.4	1.97	(1.34–2.89)
16–20	2577	1305	50.6	4.28	(2.98–6.15)
21–25	5994	4337	72.4	10.7	(7.50–15.4)
26–30	5318	4838	91.0	41.4	(28.7–59.7)
≥31	1644	1590	96.7	121	(77.4–189)

CHM, Chinese herbal medicine; CI, confidence interval; OR, odds ratio.

## Discussion

Using nationwide survey data from the 2017 TSFIE, we found a very high one-year prevalence (74.8%) of purchasing non-prescribed CHM among adults. Purchasing non-prescribed CHM was associated with sociodemographic factors, expenditures on unhealthy lifestyle habits, the use of medical care, the use of folk therapy, and the purchase of biochemical medications and health food.

Sex is an important factor associated with medical care and CAM [14]. In general, women were found to have better knowledge, attitudes, and practices regarding self- management of health than men, and they were also more willing to address health problems by trying multiple therapies, including conventional medicine, CAM, biochemical medications, health foods, and herbal medicine [14]. In addition, social networking and being a housekeepers make it easier for women than men to access non-prescribed CHM for themselves or family members [19]. Similar to previous study findings [14], our results showed that women were more likely to purchase non-prescribed CHM than men.

Compared with young people, elderly individuals have more coexisting medical conditions, and they are relatively more willing to try multiple therapies to treat their illnesses [13]. A previous study also suggested that older people are more likely to purchase non-prescribed CHM to improve their well-being and disease-related symptoms than young people [14]. The survey regarding CAM use in Tokyo showed that older people had a higher prevalence of CAM use than people aged younger than 60 years [20]. Older people are the major purchasers of TCM products not covered by Taiwan’s National Health Insurance [21]. Therefore, our finding of a higher likelihood of purchasing non-prescribed CHM among elderly individuals than among young people is reasonable.

In the present study, we found that participants who lived in highly urbanized areas had a higher likelihood of purchasing non-prescribed CHM. In general, residents in urban areas have more access to information on conventional and unconventional therapies than rural residents because there is more information on health management in cities than in rural areas [22, 23]. Our study also found that people who work in agriculture, with animals or in blue-collar jobs had a relatively high likelihood of purchasing non-prescribed CHM because many of these people live in rural areas and have low levels of education and income. These findings were consistent with those of previous studies [14].

People living in urban areas have higher levels of income than in people living in rural areas, and income is one of the determinants of purchasing CAM [2]. We hypothesized that people with high income levels would have much more money to purchase conventional medicine, traditional medicine, folk therapy, health food, and non-prescribed medicine not covered by insurance. This viewpoint also helps explain the association of purchasing of biochemical medications, purchasing health food, using of folk therapy, and high expenditure levels on alcohol were factors associated with purchasing non-prescribed CHM in this study. Having a stable income is an important factor in a marriage. We also found that being married was associated with purchasing non-prescribed CHM in this study. Caring about each other, paying more attention to each other, and expressing concern prompt people with illnesses and their family members to purchase non-prescribed CHM. A previous study indicated that marital status was a factor influencing the use of dietary supplements in South Korea [21].

Under the coverage of Taiwan’s National Health Insurance Program, people with illnesses or diseases can easily receive Western medical care and TCM. It is understandable that people with a disease or illness might try multiple therapies to improve their well-being and disease-related symptoms [14]. This may explain why the use of outpatient Western medical care and TCM were associated with the purchase of non-prescribed CHM. However, our study also showed that people who were not hospitalized had a greater likelihood of purchasing non-prescribed CHM than those who had experienced hospitalization. In general, patients who need inpatient care in the form of hospitalization have relatively more severe medical conditions than those who need only outpatient care. Hospitalized patients have less opportunity to purchase non-prescribed CHM, and most of these patients had better compliance with Western medicine inpatient care.

A Palestinian survey found that the prevalence of potential drug- herb interactions was 24.9% among 237 patients with chronic disease who had used at least one herb and that male sex, older age, a higher number of biochemical drugs, and more chronic disease were associated factors [15]. A large scale study based on data of Taiwan’s National Health Insurance suggested that Ma Huang, Dang Gui, and Baizhi were the most common Chinese herbs interacting with biochemical medicines in 1998–2011 [24]. Interactions between herbal medicines and prescribed drugs should be considered when using herbal medicine [15, 16, 24, 25].

Some limitations existed in this nationwide survey. First, due to the cross-sectional study design, we could not provide information regarding the long-term trend in purchasing non-prescribed CHM. The causal inferences between purchasing CHM and the associated factors are also limited in this study. Second, recall bias is always possible because our data were obtained with face-to-face interviews. Elderly people and those with brain-related illnesses may struggle to recall the information from the past year. Third, we have no further information regarding the dosage or frequency of use of the purchased non-prescribed CHM. Thus, we could not evaluate these details among people in Taiwan. In addition, previous studies suggested that medical conditions and underlying diseases are determinants of seeking TCM, purchasing herbal medicine, and using folk therapy [13, 14, 23, 26]. One of the study limitations is that the information regarding the history of diseases and medical conditions of participants was not available in the database of the 2017 TSFIE. Finally, we tried to use scoring model to investigate whether predictive score is associated with the purchase of non-prescribed CHM. However, we have no other database for further validation and this is one of our study limitations. Our predictive model needs further improvement, validation, and evaluation by future well-design studies.

In conclusion, a very high one-year prevalence of purchasing non-prescribed CHM was observed among adults in Taiwan, and the purchasing of non-prescribed CHM was associated with sociodemographic factors and the use of medical care. The findings of our study remind health policy makers, clinical Western medicine physicians and TCM physicians that the purchasing of non-prescribed CHM is a serious problem. The interactions of non-prescribed CHM with physician-prescribed herbal medicine and biomedical medications needs more attention.

## Supporting information

S1 Table Expenditure on non-prescribed Chinese herbal medicine by baseline characteristics (N = 12,374). (DOC)

## List of abbreviations

*ICD-9-CM*: *International Classification of Diseases*, *9th Revision*, *Clinical Modification*
CI: confidence interval
OR: odds ratio
CHM: Chinese herbal medicine

## References

[1] Clarke TC, Black LI, Stussman BJ, Barnes PM, Nahin RL. Trends in the use of complementary health approaches among adults: United States, 2002–2012. Natl Health Stat Report. 2015;79: 1–16. PMC4573565 25671660

[2] Kemppainen LM, Kemppainen TT, Reippainen JA, Salmenniemi ST, Vuolanto PH. Use of complementary and alternative medicine in Europe: Health-related and sociodemographic determinants. Scand J Public Health. 2018;46: 448–455. 10.1177/1403494817733869 28975853 PMC5989251

[3] Misawa J, Ichikawa R, Shibuya A, Maeda Y, Hishiki T, Kondo Y. Social determinants affecting the use of complementary and alternative medicine in Japan: An analysis using the conceptual framework of social determinants of health. PLoS One. 2018;13: e0200578 10.1371/journal.pone.0200578 30011303 PMC6047791

[4] Nahin RL, Barnes PM, Stussman BJ. Expenditures on complementary health approaches: United States, 2012. Natl Health Stat Report. 2016;95: 1–11. 27352222

[5] Yang L, Adams J, Sibbritt D. Prevalence and factors associated with the use of acupuncture and Chinese medicine: results of a nationally representative survey of 17161 Australian women. Acupunct Med. 2017;35: 189–199. 10.1136/acupmed-2016-011179 28279972

[6] Burke A, Upchurch DM, Dye C, Chyu L. Acupuncture use in the United States: findings from the National Health Interview Survey. J Altern Complement Med. 2006;12: 639–648. 10.1089/acm.2006.12.639 16970534

[7] Upchurch DM, Rainisch BW. A sociobehavioral wellness model of acupuncture use in the United States, 2007. J Altern Complement Med. 2014;20: 32–39. 10.1089/acm.2012.0120 23414108 PMC3904513

[8] Huang CW, Tran DNH, Li TF, Sasaki Y, Lee JA, Lee MS, et al The utilization of complementary and alternative medicine in Taiwan: an internet survey using an adapted version of the international questionnaire (I-CAM-Q). J Chin Med Assoc. 2019;82: 665–671. 10.1097/JCMA.0000000000000131 31305349 PMC13048053

[9] Chang CC, Chen TL, Chiu HE, Hu CJ, Yeh CC, Tsai CC, et al Outcomes after stroke in patients receiving adjuvant therapy with traditional Chinese medicine: a nationwide matched interventional cohort study. J Ethnopharmacol. 2016;177: 46–52. 10.1016/j.jep.2015.11.028 26593214

[10] He Y, Guo X, May BH, Zhang AL, Liu Y, Lu C, et al Clinical evidence for association of acupuncture and acupressure with improved cancer pain: a systematic review and meta-analysis. JAMA Oncol. 2019;6: 271–278. 10.1001/jamaoncol.2019.5233 PMC6990758 31855257

[11] Lu W, Giobbie-Hurder A, Freedman RA, Shin IH, Lin NU, Partridge AH, et al Acupuncture for chemotherapy-induced peripheral neuropathy in breast cancer survivors: a randomized controlled pilot trial. Oncologist. 2020;25: 310–318. 10.1634/theoncologist.2019-0489 32297442 PMC7160396

[12] Wang S, Tang Q, Qian W, Fan Y. Meta-analysis of clinical trials on traditional Chinese herbal medicine for treatment of persistent allergic rhinitis. Allergy. 2012;67: 583–592. 10.1111/j.1398-9995.2012.02806.x 22435619

[13] Liao HL, Ma TC, Chiu YL, Chen JT, Chang YS. Factors influencing the purchasing behavior of TCM outpatients in Taiwan. J Altern Complement Med. 2008;14: 741–748. 10.1089/acm.2007.7111 18684079

[14] Shih CC, Huang LH, Yeh CC, Lane HL, Hsieh CJ, Tsai CC, et al The prevalence, characteristics, and factors associated with purchasing Chinese herbal medicine among adults in Taiwan. BMC Complement Altern Med. 2017;17: 169 10.1186/s12906-017-1679-2 28347338 PMC5369211

[15] Al-Ramahi R, Jaradat N, Shalalfeh R, Nasir S, Manasra Y, Shalalfeh I, et al Evaluation of potential drug- herb interactions among a group of Palestinian patients with chronic diseases. BMC Complement Altern Med. 2015;15: 221 10.1186/s12906-015-0764-7 26162600 PMC4702382

[16] Izzo AA, Ernst E. Interactions between herbal medicines and prescribed drugs: an updated systematic review. Drugs. 2009;69: 1777–1798. 10.2165/11317010-000000000-00000 19719333

[17] Kim JH, Kwong EM, Chung VC, Lee JC, Wong T, Goggins WB. Acute adverse events from over-the-counter Chinese herbal medicines: a population-based survey of Hong Kong Chinese. BMC Complement Altern Med. 2013;13: 336 10.1186/1472-6882-13-336 24279604 PMC4222756

[18] Zhong LLD, Zheng G, Da Ge L, Lin CY, Huang T, Zhao L, et al Chinese herbal medicine for constipation: zheng-based associations among herbs, formulae, proprietary medicines, and herb-drug interactions. Chin Med. 2016;11: 28 10.1186/s13020-016-0099-4 27347002 PMC4919884

[19] Liu CY, Liu JS. Socioeconomic and demographic factors associated with health care choices in Taiwan. Asia Pac J Public Health. 2010;22: 51–62. 10.1177/1010539509352024 20032035

[20] Hori S, Mihaylov I, Vasconcelos JC, McCoubrie M. Patterns of complementary and alternative medicine use amongst outpatients in Tokyo, Japan. BMC Complement Altern Med. 2008;8: 14 10.1186/1472-6882-8-14 18433476 PMC2375857

[21] Ock SM, Hwang SS, Lee JS, Song CH, Ock CM. Dietary supplement use by South Korean adults: data from the national complementary and alternative medicine use survey (NCAMUS) in 2006. Nutr Res Pract. 2010;4: 69–74. 10.4162/nrp.2010.4.1.69 20198211 PMC2830417

[22] Blenkinsopp A, Bradley C. Patients, society, and the increase in self medication. BMJ. 1996;312: 629–632. 10.1136/bmj.312.7031.629 8595343 PMC2350384

[23] Shih CC, Huang LH, Lane HL, Tsai CC, Lin JG, Chen TL, et al Use of folk therapy in Taiwan: a nationwide cross-sectional survey of prevalence and associated factors. Evid Based Complement Alternat Med. 2015;2015: 649265 10.1155/2015/649265 26170878 PMC4480812

[24] Chen KC, Lu R, Iqbal U, Hsu KC, Chen BL, Nguyen PA, et al Interactions between traditional Chinese medicine and western drugs in Taiwan: A population-based study. Comput Methods Programs Biomed. 2015;122: 462–70. 10.1016/j.cmpb.2015.09.006 26470816

[25] Zuo Z, Huang M, Kanfer I, Chow MS, Cho WC. Herb-drug interactions:systematic review, mechanisms, and therapies. Evid Based ComplementAlternat Med. 2015;2015:239150. 10.1155/2015/239150 PMC4352432 25792995

[26] Weng SW, Chen TL, Yeh CC, Liao CC, Lane HL, Lin JG, et al An investigation of the use of acupuncture in stroke patients in Taiwan: a national cohort study. BMC Complement Altern Med. 2016;16: 321 10.1186/s12906-016-1272-0 27566677 PMC5002127

