# Supplementary material for: Prevalence, expenditures, and associated factors of purchasing non-prescribed Chinese herbal medicine in Taiwan
Source: PLoS One. 2020 Oct 26;15(10):e0240311. doi: 10.1371/journal.pone.0240311 (PMC7588068; doi:10.1371/journal.pone.0240311)
Supplement: S1 Table — (DOC) [file pone.0240311.s001.doc]

| **S1 Table.** Expenditure on non-prescribed Chinese herbal medicine by baseline characteristics (N=12,374) | | | | | | |
| --- | --- | --- | --- | --- | --- | --- |
|  | | CHM purchase | | | |  |
|  | | n | Mean | ± | S.D. | *p*-Value |
| Age, years | 18-29 | 468 | 77 | ± | 163 | 0.7355 |
|  | 30-39 | 2056 | 79 | ± | 238 |  |
|  | 40-49 | 2853 | 73 | ± | 122 |  |
|  | 50-59 | 3161 | 75 | ± | 131 |  |
|  | 60-69 | 2303 | 72 | ± | 145 |  |
|  | 70-97 | 1531 | 72 | ± | 155 |  |
| Sex | Female | 3542 | 68 | ± | 138 | 0.0030 |
|  | Male | 8832 | 77 | ± | 165 |  |
| Occupation | None | 2271 | 66 | ± | 139 | <0.0001 |
|  | White collar | 1739 | 94 | ± | 263 |  |
|  | Senior technician | 2573 | 73 | ± | 112 |  |
|  | Services | 1739 | 79 | ± | 167 |  |
|  | Agriculture/animal | 676 | 66 | ± | 107 |  |
|  | Blue collar | 1853 | 70 | ± | 141 |  |
|  | Others | 1523 | 71 | ± | 113 |  |
| Education, years | 0 | 242 | 57 | ± | 121 | 0.0070 |
|  | 1-9 | 7343 | 72 | ± | 171 |  |
|  | 10-12 | 4030 | 77 | ± | 137 |  |
|  | ≥13 | 759 | 88 | ± | 146 |  |
| Marital status | Unmarried | 802 | 65 | ± | 152 | <0.0001 |
|  | Married | 8821 | 79 | ± | 168 |  |
|  | Other | 2751 | 64 | ± | 122 |  |
| Dental care | No | 2166 | 70 | ± | 125 | 0.1190 |
|  | Yes | 10208 | 75 | ± | 164 |  |
| Use of WM | No | 112 | 65 | ± | 114 | 0.5369 |
|  | Yes | 12262 | 75 | ± | 159 |  |
| Use of TCM | No | 6026 | 67 | ± | 137 | <0.0001 |
|  | Yes | 6348 | 82 | ± | 176 |  |
| Hospitalization | No | 11030 | 72 | ± | 152 | <0.0001 |
|  | Yes | 1344 | 97 | ± | 201 |  |
| Medication purchase | No | 691 | 83 | ± | 167 | 0.1516 |
|  | Yes | 11683 | 74 | ± | 158 |  |
| Use of folk therapy | No | 10571 | 71 | ± | 163 | <0.0001 |
|  | Yes | 1803 | 92 | ± | 127 |  |
| Health food purchase | No | 1251 | 63 | ± | 183 | 0.0069 |
|  | Yes | 11123 | 76 | ± | 155 |  |
| Level of income | Low | 4480 | 54 | ± | 104 | <0.0001 |
|  | Moderate | 2529 | 65 | ± | 107 |  |
|  | High | 5365 | 96 | ± | 206 |  |
| Smoking expenditure | No | 8963 | 73 | ± | 159 | 0.0851 |
|  | 1-300 | 571 | 80 | ± | 143 |  |
|  | 301-600 | 1027 | 74 | ± | 151 |  |
|  | 601-900 | 627 | 73 | ± | 128 |  |
|  | ≥900 | 1186 | 86 | ± | 177 |  |
| Alcohol expenditure | No | 967 | 69 | ± | 141 | <0.0001 |
|  | 1-30 | 4871 | 60 | ± | 182 |  |
|  | 31-60 | 1793 | 72 | ± | 130 |  |
|  | 61-150 | 2716 | 81 | ± | 136 |  |
|  | ≥150 | 2027 | 105 | ± | 152 |  |
| Urbanization | Low | 1595 | 70 | ± | 157 | 0.0006 |
|  | Moderate | 4833 | 69 | ± | 173 |  |
|  | High | 5946 | 80 | ± | 145 |  |
| CHM, Chinese herbal medicine; TCM, traditional Chinese medicine; WM, Western medicine | | | | | | |
